# Complications of Trauma Patients Admitted to the ICU in Level I Academic Trauma Centers in the United States

**DOI:** 10.1155/2014/473419

**Published:** 2014-06-03

**Authors:** Stefania Mondello, Amy Cantrell, Domenico Italiano, Vincenzo Fodale, Patrizia Mondello, Darwin Ang

**Affiliations:** ^1^Department of Neurosciences, University of Messina, Via Consolare Valeria, 98125 Messina, Italy; ^2^Department of Biostatistics, College of Public Health and Health Professions, College of Medicine, University of Florida, P.O. Box 117450, 2004 Mowry Road, 5th Floor CTRB, Gainesville, FL 32611-7450, USA; ^3^Department of Clinical and Experimental Medicine and Pharmacology, University of Messina, Via Consolare Valeria, 98125 Messina, Italy; ^4^Department of Human Pathology, University of Messina, Via Consolare Valeria, 98125 Messina, Italy; ^5^University of South Florida and Trauma Services, Ocala Regional Medical Center, Ocala, FL, USA

## Abstract

*Background*. The aims of this study were to evaluate the complications that occur after trauma and the characteristics of individuals who develop complications, to identify potential risk factors that increase their incidence, and finally to investigate the relationship between complications and mortality. *Methods*. We did a population-based retrospective study of trauma patients admitted to ICUs of a level I trauma center. Logistic regression analyses were performed to determine independent predictors for complications. *Results*. Of the 11,064 patients studied, 3,451 trauma patients developed complications (31.2%). Complications occurred significantly more in younger male patients. Length of stay was correlated with the number of complications (*R* = 0.435, *P* < 0.0001). The overall death rate did not differ between patients with or without complications. The adjusted odds ratio (OR) of developing complication for patients over age 75 versus young adults was 0.7 (*P* < 0.0001). Among males, traumatic central nervous system (CNS) injury was an important predictor for complications (adjusted OR 1.24). *Conclusions*. Complications after trauma were found to be associated with age, gender, and traumatic CNS injury. Although these are not modifiable factors, they may identify subjects at high risk for the development of complications, allowing for preemptive strategies for prevention.

## 1. Introduction

Trauma is a major health problem and a leading cause of mortality and morbidity among young individuals in the world [1]. In the United States, Centers for Disease Control and Prevention reported that more than 50 million patients receive medical care for trauma annually and approximately 30 percent of all intensive care unit admissions are a consequence of a trauma [2, 3]. The range of injury is broad and heterogeneous, from severe injuries involving multiple organ systems to isolated extremity wounds. In the United States trauma is responsible for 10 percent of all deaths [4], but changes in the trauma epidemiology with gradual improvement in mortality rates have been reported [5–8]. The changing pattern of traumatic death has been related to several causes such as improvements in surgical techniques and diagnostics, implementation of advanced trauma life support (ATLS), patient management, and treatment strategies [9]. The ability to keep severely injured trauma patients alive has resulted in an increased incidence of complication in this population [8]. Complications that occur in trauma patients are associated with increased morbidity, length of stay, and possible late death and are also responsible for a significant financial cost [2, 10].

It is not currently possible to reliably predict the occurrence, timing, or type of complications in individual patients. However, identifying the subgroup(s) of patients (risk factors) that may develop complications may allow for preemptive rather than reactive therapy. In addition, identification of the epidemiology, patterns, and causes of complications following trauma may provide useful information for improving treatment strategies, outcomes, and costs ultimately enhancing the quality of the health system, especially in the area of trauma care (Level I Trauma Center) [11]. The aims of this study were to evaluate the incidence and type of complications that can occur after trauma among patients critically ill enough to be admitted to the ICU and to determine the independent predictors of complications and mortality.

## 2. Materials and Methods

### 2.1. Study Population

This study is a population-based retrospective cohort study. that was conducted using data from the University HealthSystem Consortium (UHC), an alliance of over 90% of academic medical centers and their affiliated hospitals in the United States [12]. The UHC database is a large administrative data set encompassing information on all hospital discharges in the consortium including patient demographics, discharge diagnoses, and outcomes.

This study included 11064 patients, 18 years of age or older, presenting with trauma and admitted to an intensive care unit (ICU) from May 2008 to April 2009. Trauma characteristics of patients were identified by selecting a specific group of ICD-9-Clinical Modification (ICD-9-CM) codes defined by the American College of Surgeons (ACS). Demographic and clinical data, including age, sex, mechanism of injury, procedures, hospital length of stay (LOS), complications, and inhospital mortality were obtained. For the purpose of the analysis, cause of admission was grouped into 4 major categories: internal injury (24.2%), traumatic CNS injury (23.7%), fracture (16.8%), and others (35.3%). All patients in our study had an invasive procedure performed such as surgery or vascular catherization.

The overall number of complications per patient was identified as well as the presence or absence of specific complications. A standardized manual for definitions of complications was used for reference [13]. This study reported 23 types of complications, three of which did not have enough events for meaningful statistical analysis.

The primary outcome of interest was presence or absence of complications. The secondary outcome of interest was mortality.

### 2.2. Statistical Analysis

Exploratory analysis was carried out to determine the distribution of the demographic and clinical variables. Continuous variables are presented as mean (SD) or median (interquartile range). Distributions of categorical variables were presented as frequencies and percentages. The association between each continuous variable and complications or mortality was evaluated using the Mann-Whitney *U* test (2 groups) or the Kruskal-Wallis test (3 or more groups). The association between each categorical variable and complications or mortality was evaluated using the chi-square or Fisher's exact test, as indicated.

Univariate logistic regression analysis was used to evaluate the prognostic ability of the demographic and clinical variables, individually, to predict the probability of development of complications or death. Crude odds ratios with 95% confidence intervals are presented. The *R*-squared is given of each model to indicate the percentage of variation in the outcome that can be explained by the variable. Because age and LOS were found to be nonlinearly related to the log-odds for each outcome; both variables were categorized. Age was categorized into young adults (18–44), middle aged (45–64), elderly (65–74), and advanced seniority (≥75). LOS was divided into equal tertiles according to the 33rd and 77th percentile. Patients were categorized on the basis of LOS into lower (<5 days), middle (5–19 days), and high (≥20 days) tertiles.

Variables associated with each outcome in the univariate analysis (*P* < 0.05) were included in additional multivariable logistic regression models. Logistic regression analysis was performed to determine factors that could be considered independent risk factors for complications and mortality; adjusted odds ratios are reported with their respective 95% CIs. We evaluated several models due to collinearity of candidate variables. For additional validation of the model selected, we also used forward stepwise selection with an inclusion criterion *P* value of 0.10 (the variable was added to the model if the corresponding *P* value was less than the defined threshold 0.10. Otherwise, the variable was not considered sufficiently useful to enter the model). In addition, we explored the interactions. Interaction terms were investigated by including them individually in the candidate models.

All hypothesis tests conducted were 2-tailed. A *P* value < 0.05 was considered significant. All statistical analyses were performed using SAS (SAS version [9.2] of the SAS System. Copyright © 2002–2008 by SAS Institute Inc., Cary, NC, USA).

## 3. Results

### 3.1. Population Characteristics and Outcome

The demographic and clinical data are summarized in Table 1. Age was significantly different in males compared to females (*P* < 0.0001), with females tending to be older than males (median 51.7 versus 43.6 years). There was also a significant difference in age between deceased individuals and survivors (*P* < 0.0001), with deceased individuals tending to be older than survivors (median 55.9 versus 44.1 years). There was a significant difference in the mortality rate between males and females (17.36% versus 19.46%, *P* = 0.01).

### 3.2. Complications Characteristics

Among patients studied, 31.2% developed complications. The characteristics of patients with and without complications are listed in Table 2. Specific complication frequencies and percentages are presented in Table 3. There was a significant difference in age between the two groups (median, 46.3 versus 45.2, *P* = 0.0009). Complications occurred more frequently in males than females (32.5% versus 27.96%, *P* < 0.0001). The mortality was not significantly different between patients with and without complications (*P* = 0.52), while there was a highly significant difference in LOS between subjects with and without complications (median, 6 versus 18 days, respectively, *P* < 0.0001). LOS was highly correlated with number of complications (Spearman's rank correlation coefficient = 0.435, *P* < 0.0001). Age was not associated with LOS.

Most patients had a single complication (60.8%) (Table 1). Postoperative pulmonary compromise was the most frequent complication (1733; 30.7%) (Table 3). The percentage of postoperative pulmonary compromise was significantly higher among subjects who died (43.8%) compared to subjects who survived (27.9%) (*P* < 0.0001). Other complications which were significantly more common in subjects who died were postoperative cardiac abnormality (3.33% versus 0.30%, *P* < 0.0001), shock or cardiorespiratory arrest (1.61% versus 0.21%, *P* < 0.0001), and postoperative AMI (2.22% versus 0.84%, *P* = 0.0004). Complications which were more common in survivors were venous thrombosis/pulmonary embolism (12.19% versus 5.95%, *P* < 0.0001), cellulitis or decubitus ulcer (6.57% versus 2.32%, *P* < 0.0001), wound infection (5.19% versus 2.02%, *P* < 0.0001), other complications of procedures (8.82% versus 5.35%, *P* = 0.0002), and postoperative infections that are not pneumonia/wound (3.67% versus 1.61%, *P* = 0.001) (Table 3). The prevalence of other complications was not significantly different between survivors and nonsurvivors.

Eight hundred and four patients (23.3%) developed two complications. Interestingly, more than 50% of the patients with 2 complications presented a pulmonary complication.

### 3.3. Univariate Analysis and Multiple Logistic Regression Analysis for Complications

Individual logistic regression models examining the strength of association between each clinical and demographic variable and the development of complications were constructed. This analysis showed that several characteristics predict complication after trauma (Table 4). After categorizing age in a univariate analysis, patients in the oldest group (advanced seniority, >75) are estimated to have 32% lower odds of developing complications than patients in the young adult group (18–45) (OR 0.68, 95% CI 0.60 to 0.77, *P* < 0.0001). Patients in the middle age (46–59) and elderly (60–74) groups did not show significant difference compared to the young adult group. Being male and having a CNS injury were both positively associated with complications (OR 1.25, 1.42–1.37, *P* < 0.0001 and OR 1.16, 1.160–1.273, *P* = 0.001, resp.) (Table 4).

Forward stepwise logistic regression analysis identified patient age, gender, and presence of CNS injury as predictors for complications (see Methods section). When we explored interactions, we found that the interaction between CNS injury and gender was also significant. Our final model included patient characteristics age and gender as well as the presence of CNS injury and the interaction between CNS injury and gender as covariates. Adjusting for all other variables in the model, analysis of complications demonstrated that patients in the advanced seniority age group have odds of developing complications which are 30% less than that among young adults (adjusted OR 0.70, 95% CI 0.61 to 0.80, *P* < 0.0001). Among males, presence of a CNS injury was positively associated with complications compared to those without a CNS injury (adjusted OR 1.24, 95% CI 1.1 to 1.38), whereas there was no significant difference among females.

### 3.4. Univariate and Multivariate Analysis for Mortality Characteristics

Univariate binary logistic regression analysis showed that several characteristics were strongly associated with death after trauma (Table 5). Each one year increase in age was associated with a 2% increase in the odds of mortality (OR 1.02; 95% CI 1.022–1.027, *P* < 0.0001). After categorizing age, in a univariate analysis with young adults (aged between 18 and 45) as the reference, there is an increasing trend in the odds of mortality with increasing age (Table 5). Being male was associated with a decreased mortality (OR 0.869, 95% CI 0.783–0.966). Patients with traumatic CNS injury showed higher risk of mortality (OR 4.549, 95% CI 4.106–5.040). Presence of complications and number of complications were not associated with an increased risk of mortality (*P* = 0.52 and *P* = 0.52, resp.).

Forward stepwise logistic regression analysis, including patient characteristics (age, gender) and trauma characteristics (diagnosis on admission) as covariates, identified patient age and diagnosis on admission as predictors of death. Complementary to the previous model, stepwise logistic regression analysis, including patient characteristics (age, gender) and traumatic CNS injury as covariates, identified patient age and traumatic CNS injury as predictors of death. Gender was not significant in the full model. It is likely that the gender's effect in the simple model was related to age. No significant interactions were found. In multivariate analysis of young adults with no traumatic CNS injury as the references, patients in advanced seniority (adjusted OR 4.30, 95% CI 3.72–4.97, *P* < 0.0001) showed a higher odds ratio compared to those in elderly (adjusted OR 2.15, 95% CI 1.87–2.50, *P* < 0.0001) and middle aged (adjusted OR 1.41, 95% CI 1.23–1.62, *P* < 0.0001). Having traumatic CNS injury was a strong independent predictor of death (adjusted OR 4.74, 95% CI 4.27–5.27, *P* < 0.0001).

## 4. Discussion

Complications following admission for traumatic injury are common and have been shown to increase morbidity, length of stay, and costs in a level I trauma center [10, 14, 15] as well as to have a negative impact on long-term quality of life of trauma patients [16]. Evaluating complications and their risk factors is therefore essential to enhance adoption of best practices to reduce complications that will lead to improve outcome, resource utilization, and quality of care for trauma patients.

The objective of this study was to describe epidemiologic features, risk factors for acquisition, and outcome of complications that can occur after trauma in a cohort of 11,064 patients who were admitted to the ICU in Level I Academic Trauma Centers. The findings from our study show that (1) age, gender, diagnosis on admission, and CNS injury were associated with higher incidence of complications; (2) occurrence and number of complications correlated with LOS but not with mortality; and (3) mortality and complications are associated with different risk factors.

Our data suggest that there is a gender-related difference in complication rates. In particular, we demonstrated that male patients had substantially higher incidences of complications. Supporting these observations, multivariable logistic regression analysis identified gender as an independent predictor, with men exhibiting higher odds of developing complications when compared to female patients. In line with our findings, there is an increasing number of experimental and human studies supporting a gender-related differences among trauma patients in developing complications [17, 18]. Recently, an analysis of prospectively collected data from adult trauma patients admitted to hospitals in the National Trauma Data Bank has shown that women are less likely than men to develop inpatient complications [19]. Similarly, in the largest single-institution series of blunt trauma patients including >36,000 patients, male gender was shown to be associated with increased morbidity [18]. It is not clear why women appear to be less susceptible to developing complications than men; most investigators agree that these differences are due to both a deleterious effect of testosterone [20] and a beneficial effect of female sex hormones estrogens conferring an immunoenhancing effect and therefore protection [21].

Additionally, an interaction analysis was undertaken to evaluate whether gender impacts the association between complications and CNS injury. The results of our analysis showed that if a patient sustained a traumatic CNS injury, there were predictive gender differences. Men having a traumatic CNS injury were found to have a 24% higher odd of developing complications over those without a CNS injury. In contrast, the presence of CNS injury showed no significant increase in the odds of complications as compared to females without CNS injury. This finding supports previous experimental and clinical evidence showing gender-related differences in outcome after a neurotrauma [22, 23] and the hormonal influence and neuroprotective effects of sex hormones in injured brain [24–26]. The concept of examining the impact of gender on complications in trauma patients with a CNS injury is novel and might suggest new therapeutic approaches. However, further studies are needed to confirm and assess the role of gender as modulator of the incidence of complications after traumatic CNS injury.

Another interesting finding is that, despite this gender-related difference in developing complications, there was no difference in survival which is consistent with epidemiological studies and clinical experience [18]. This observation is extremely valuable by highlighting the need for identifying and selecting appropriate outcomes for clinical trials of patients with trauma and might provide a guide for their successful implementation.

In line with previous research [15] we found a correlation between occurrence of complications and LOS but not with mortality. The lack of relationship between complications and mortality is conceivable. As the majority of trauma deaths occur in the first 24 hours, patients who die have the shortest LOS. It is also possible that the lack of direct correlation between mortality and complications may be related to the advances in medicine. Advances in prehospital care, as well as in medical and surgical care, may be responsible for the decrease in mortality rates of trauma patients allowing more severely injured patients to survive [6, 8, 27]. The results for these advances may produce a more fragile population susceptible and vulnerable to the risk of complications among those who could survive the initial insult. These findings have several important implications. First, salvage of patients who have more severe injuries may increase the complications' rates leading to prolonged hospitalization and higher costs. On the other hand, complications are potentially modifiable; therefore, implementation of practice guidelines to reduce complications could improve LOS and impact health care costs. Taken together, these observations suggest that complications could be used as a marker for quality of care and resource utilization at trauma centers. Recent studies have just started exploring this field [15].

Interestingly, consistent with previous reports [28], in our study older trauma patients have higher mortality than younger patients, whereas, the opposite was true for developing complications after trauma. One possible explanation is that the higher mortality in the older group may actually reduce the probability that this group develops complications. On average, older critically ill patients may die before complications occur. This conclusion is also supported by the correlation between LOS and complications. Another explanation could be that of natural selection or selection bias. In other words older people who survived the traumatic events were either healthier to begin with or could potentially have suffered a less severe trauma but were admitted to the ICU due to their age. On the other hand, younger patients who could survive more severe injuries spent more time in the ICU and increased their exposure for developing complications.

Not all variables were diametrically opposite for prediction of either complications or mortality. For example, traumatic CNS injury was significantly associated with both complications and mortality. Traumatic CNS injury has been demonstrated a primary cause of death in previous reports [29, 30].

It should also be noted that when stratifying complication type with mortality, complications related to a cardiopulmonary process were more significantly associated with mortality; while blood borne complications such as infection and deep vein thrombus were more associated with those who survived. While the severity of some complications are more lethal, the frequency of nonlethal complications account for the majority of ICU complications (Table 3).

Our study has some limitations. The uncertainty about the timing of complication onset did not allow us to investigate the temporal distribution of the events. In particular, we were not able to establish a temporal relationship (and potentially causal-effect relationship) between complications and mortality. However, the intent of our studies was to investigate the general features of complications in trauma population and to compare risk factors associated with the onset of complications with those associated with death. An additional constraint was the use of an administrative database. Administrative databases are an important source of information, and they are especially convenient in studying low frequency events. However, their main limitation is related to the level of detail required for clinical interventions at the bedside for the different conditions and diseases especially in an ICU setting [31, 32].

A main strength of this article is that it represents one of the largest groups of ICU patients in which complications were evaluated. This provides the statistical power to capture even rare clinical events. Furthermore, this is a multicenter study representing over 90% of academic centers in the United States. This avoids the bias that can be present in studies using a single center. Additionally, our study includes Level 1 trauma centers that consistently provide the highest level of surgical care and ICU management and a full spectrum of patients. Therefore, these results should be representative and can be extrapolated towards the general population of trauma patients.

## 5. Conclusions

The current research of >11,000 patients has provided characterization of patients and their complications which develop after trauma. This valuable information may help identify subjects at high risk for the future development of complications and be applied in clinical practice for preemptive strategies. Furthermore, using multivariable logistic regression analysis we have shown that complications and mortality among ICU trauma patients are associated with different risk factors. This means that modifying factors influencing occurrence of complications do not necessarily offer survival advantage after trauma. Therefore, before embarking on large expensive clinical trials targeting or manipulating specific variables, it is of paramount importance to conduct thorough studies adequately addressing the role and interactions of various risk factors.

## Figures and Tables

**Table 1:** Summary of demographic and clinical data for trauma cases.

Age, years	
Median (Q1–Q3)	45.96 (29.3–61.7)
Min–Max	18–94
Gender, *n* (%)	
Female	3196 (28.9%)
Male	7868 (71.1%)
Mortality, *n* (%)	
Alive	9076 (82.0%)
Dead	1988 (18.0%)
Complication, *n* (%)	
No	7613 (68.8%)
Yes	3451 (31.2%)
Number of complications, *n* (%)	
1	2097 (60.8%)
2	804 (23.3%)
3	349 (10.1%)
4	132 (3.8%)
5	47 (1.4%)
6	21 (0.61%)
9	1 (0.03%)

**Table 2:** Characteristics of patients with and without complications.

Characteristics		No complications (*n* = 7613)	Complications (*n* = 3451)	*P* value^a^
Age:	Median (Q1–Q3)	46.33 (29.58–62.67)	45.17 (28.83–60)	0.0009**
Gender: F/M	*n* (%)	2306/5307 (30/70)	890/2561 (26/74)	<0.0001***
Mortality: No/Yes	*n* (%)	6257/1356 (82/18)	2819/632 (82/18)	0.52

^a^Mann-Whitney *U* test for age and Fisher's exact test for gender and mortality.

Significant differences are indicated with *(*P* < 0.05),**(*P* < 0.001) or ***(*P* < 0.0001).

**Table 3:** Summary of complications for trauma cases.

Complication	Overall *N* (%)	Survivors *N* (%)	Deceased *N* (%)	*P* value
Postoperative pulmonary compromise	1733 (30.7%)	1299 (27.9%)	434 (43.8%)	<0.0001***
Venous thrombosis/pulmonary embolism	627 (11.1%)	568 (12.2%)	59 (6.0%)	<0.0001***
Other complications of procedures	464 (8.2%)	411 (8.8%)	53 (5.4%)	0.0002**
Mechanical complications due to device or implant	356 (6.3%)	306 (6.6%)	50 (5.1%)	0.0588
Cellulitis or decubitus ulcer	329 (5.8%)	306 (6.6%)	23 (2.3%)	<0.0001***
Postprocedural hemorrhage or hematoma	312 (5.5%)	261 (5.6%)	51 (5.2%)	0.4951
Postoperative pneumonia	309 (5.5%)	244 (5.2%)	65 (6.6%)	0.1772
Reopening of surgical site	262 (4.6%)	218 (4.7%)	44 (4.4%)	0.6748
Wound infection	262 (4.6%)	242 (5.2%)	20 (2.0%)	<0.0001***
Miscellaneous complications	254 (4.5%)	228 (4.9%)	26 (2.6%)	0.0016*
Procedure-related perforations or lacerations	192 (3.4%)	155 (3.3%)	37 (3.7%)	0.7043
Postoperative infections not pneumonia/wound	187 (3.3%)	171 (3.7%)	16 (1.6%)	0.0010*
Postoperative GI hemorrhage or ulceration	84 (1.5%)	71 (1.5%)	13 (1.3%)	0.6494
Postoperative stroke	82 (1.5%)	67 (1.4%)	15 (1.5%)	1.000
Postoperative AMI	61 (1.1%)	39 (0.84%)	22 (2.2%)	0.0004**
Postoperative cardiac abnormality	47 (0.83%)	14 (0.30%)	33 (3.3%)	<0.0001***
Shock or cardiorespiratory arrest	26 (0.46%)	10 (0.21%)	16 (1.6%)	<0.0001***
Aspiration pneumonia	24 (0.42%)	20 (0.43%)	4 (0.40%)	1.000
Postoperative urinary tract complication	18 (0.32%)	14 (0.30%)	4 (0.40%)	0.8703
Postoperative physical and metabolic derangements	15 (0.27%)	11 (0.24%)	4 (0.40%)	0.5881
Central or peripheral nervous system	3 (0.05%)	2 (0.04%)	1 (0.10%)	1.000
Septicemia	2 (0.04%)	1 (0.02%)	1 (0.10%)	0.7956
Complications related to anesthetic agents/CNS agents	1 (0.02%)	1 (0.02%)	0 (0.00%)	1.000

TOTAL	5650^†^	4659	991	

^†^Patients can develop more than one complication.

Significant differences are indicated with *(*P* < 0.05), **(*P* < 0.001) or ***(*P* < 0.0001).

**Table 4:** Unadjusted odds ratios of clinical and demographic characteristics for predicting complications in trauma patients, using univariate logistic regression.

Variable	OR (95% CI)	*P* value	*R* ^2^, %
Age group			5
Young adults	Reference		
Middle age	1.04 (0.95–1.15)	0.40	
Elderly	1.02 (0.91–1.15)	0.71	
Advanced seniority	0.68 (0.59–0.77)	<0.0001***	
Gender			30
Female	Reference		
Male	1.25 (1.37–1.42)	<0.0001***	
Traumatic CNS injury			10
No	Reference		
Yes	1.161 (1.160–1.273)	0.001*	
Diagnosis on admission			2
Other	Reference		
Fracture	1.179 (1.012–1.374)	0.035*	
Internal injury	1.350 (1.172–1.556)	<0.0001***	
Traumatic CNS injury	1.306 (1.132–1.506)	0.0002**	

*P < 0.05, **P < 0.001, ***P < 0.0001.

OR = odds ratios.

**Table 5:** Unadjusted odds ratios of clinical and demographic characteristics for prediction of death in trauma patients, using univariate logistic regression.

Variable	OR (95% CI)	*P* value	*R* ^2^, %
Age	1.024 (1.022–1.027)	<0.0001***	57
Age group			60
Young adults	Reference		
Middle age	1.36 (1.20–1.55)	<0.0001***	
Elderly	2.01 (1.76–2.33)	<0.0001***	
Advanced seniority	4.02 (3.51–4.60)	<0.0001***	
Gender			10
Female	Reference		
Male	0.869 (0.783–0.966)	0.009*	
Traumatic CNS injury			12
No	Reference		
Yes	4.549 (4.106–5.040)	<0.0001***	
Complications			1
No	Reference		
Yes	0.967 (0.871–1.073)	0.52	0.1
Number of complications	0.983 (0.933–1.036)	0.52	
Diagnosis on admission			15
Other	Reference		
Fracture	0.351 (0.277–0.444)	<0.0001***	
Internal injury	0.578 (0.478–0.699)	<0.0001***	
Traumatic CNS injury	3.314 (2.816–3.900)	<0.0001***	

*P < 0.05, **P < 0.001, ***P < 0.0001.

OR = odds ratios.
